# P-1778. Factors Underlying Infection-Related Mortality: Learnings from an Academic Institution with Antimicrobial Stewardship Practices

**DOI:** 10.1093/ofid/ofae631.1941

**Published:** 2025-01-29

**Authors:** Aleena Issac, V Sreeraj, Deljo Puthoor, K Adarsh A, Tabitha Merium Sabu, V K Prathibha, V Geethalakshmi, Maridas Tom Thomas, Thomas Joseph, Gopika P Mohan, Mariya Johnson, Alga P Thomas, Sneha Santhosh, P R Lubaina

**Affiliations:** Amala Institute of medical sciences Thrissur , Pathanamthitta, Kerala, India; Amala Institute of Medical Sciences, Thrissur, Kerala, India; Amala Institute of Medical Sciences, Thrissur, Kerala, India; Amala Institute of Medical Sciences, Thrissur, Kerala, India; Amala Institute of Medical Sciences, Thrissur, Kerala, India; Amala Institute of Medical Sciences, Thrissur, Kerala, India; Amala Institute of Medical Sciences, Thrissur, Kerala, India; Amala Institute of Medical Sciences, Thrissur, Kerala, India; Amala Institute of Medical Sciences, Thrissur, Kerala, India; Amala Institute of Medical Sciences, Thrissur, Kerala, India; Amala Institute of Medical Sciences, Thrissur, Kerala, India; Amala Institute of Medical Sciences, Thrissur, Kerala, India; Amala Institute of Medical Sciences, Thrissur, Kerala, India; Amala Institute of Medical Sciences, Thrissur, Kerala, India

## Abstract

**Background:**

Limited research has explored the efficacy of Antimicrobial Stewardship (AMS) interventions in reducing Infection-Related Mortality (IRM). This study aims to evaluate the impact of AMS practices on IRM and its associated factors.

**Figure:**
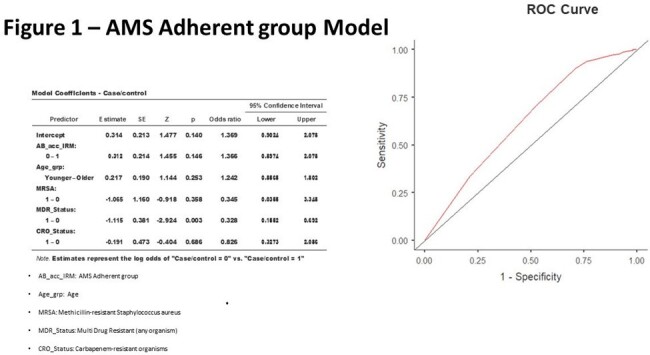

**Methods:**

A retrospective case-cohort study was conducted on inpatients who expired during hospitalization between May 1, 2022, and April 30, 2023, in a tertiary hospital. The case group comprised patients with infection-related mortalities, defined as deaths caused by any potential life-threatening infection, such as sepsis, pneumonia, infective endocarditis, peritonitis, encephalitis, abscesses, meningitis, empyema, pyelonephritis, and cholecystitis. Patients who expired without such infections constituted the control group. The primary exposure variable was adherence to AMS practices during the hospital stay. A Binomial Logistic Regression model was employed to identify risk factors (e.g., age, sex, recent multi-drug-resistant organism culture positivity) for infection-related mortality in both AMS-adherent and non-adherent groups.

**Figure:**
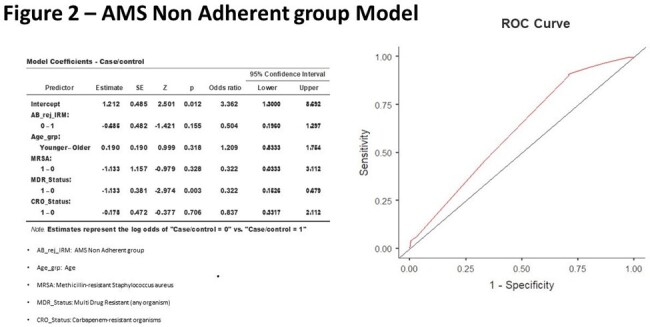

**Results:**

Among the 1225 total mortalities, 41.3% (=506) represents the case group. It was found that only recent multi-drug resistant organism culture positivity has a significant association with AMS adherence (odds ratio =0.328 & p =0.003). The statistical modeling of both groups depicted in figure 1&2.

**Conclusion:**

Despite adherence to AMS practices, the presence of multi-drug-resistant organism culture positivity predisposes individuals to infection-related mortality.

**Disclosures:**

**All Authors**: No reported disclosures

